# Medicine

**Published:** 1896-12

**Authors:** J. Michell Clarke, G. A. Bannatyne


### Progress of the Medical Sciences.

### MEDICINE.

The following is an account of some of the work done in **Serumtherapy** during the past year:

Sufficient time has now elapsed and a sufficiently large number of cases of **diphtheria** have been treated by antitoxin to put us in a better position to judge of the value of the treatment in this disease. Reports from so many different sources speak with so great unanimity with regard to the reduction in the death-rate and mitigation in the symptoms of diphtheria, that there is no room for doubt as to the superiority of this method of treatment over those formerly in vogue. This reduction in the death-rate is too great and too uniform in the most widely separated countries to be accounted for by a supposed milder type of diphtheria in the last few years, or by the fact that, as Dr. Purjesz points out,[1] the general interest taken in the antitoxin treatment by the lay papers and the public has led to a large number of mild cases being brought to the hospitals for treatment that would otherwise never have come to them. The statistics may also have been somewhat too favourable for the serum, as compared with the previous treatment, on account of the fact that the now common bacteriological examination often shows the presence of the diphtheria bacillus in cases that would have formerly been regarded as tonsillitis, and that these mild cases are injected with antitoxin. But this would not affect more than a small proportion of the cases. At the same time, antitoxin does not cure all cases of diphtheria, nor would it be reasonable to expect it to do so.

The report of the American Pediatric Society[2] is based upon the collective returns from 613 physicians, of whom over 600 pronounce themselves strongly in favour of the serum treatment. 3384 cases are reported: the doubtful cases which died are included in, those which recovered are excluded from, the report. On account of the widely separated localities from which the records came, no local peculiarities can account for the favourable results. No new cases of sudden death immediately after the injection have occurred. In nineteen cases only, injected reasonably early, the serum did not appear to have a favourable influence; of these, in nine the diagnosis was doubtful, three were malignant, four complicated with measles, and in two the serum was of doubtful value. In three the patients were made worse, but in only one of these could the result be fairly attributed to the injection. The general percentage mortality was 12.3; in cases injected during the first three days, 7.3; excluding cases moribund at the time of injection or dying within twenty-four hours, it was 8.8 and 4.8 in the two groups respectively; in cases injected on or after the fourth day it was 27. Of the laryngeal cases, many severe, one half recovered without operation. In intubated cases the mortality was 25.9 per cent., a reduction of more than 50 per cent. on any other method of treatment. Broncho-pneumonia occurred in 5.9 per cent. In contrast to two or three instances in which the serum is believed to have acted unfavourably on the heart are a large number in which the heart's action was distinctly improved. There is

[1] *Wien. med. Presse*, 1896, Nos. 25 and 26; abstract in *Centralbl. f. innere Med.*, 1896, xvii. 1145.

[2] *Pediatrics*, 1896, ii. 97; abstract in *Am. J. M. Sc.*, 1896, cxii. 214.

little, if any, evidence to show that nephritis occurred in any case as the result of the injections. Paralytic sequelæ occurred in 9.7 per cent., the effect of the serum being less marked upon the nervous system than on any other part of the body. Injection should be made as early as possible on the clinical diagnosis, not waiting for the bacteriological confirmation; but however late the first observation is made, an injection should be given unless the progress of the case is satisfactory. In severe cases, or in laryngeal diphtheria in children over two years, an initial dose of 1,500 to 2,000 units is sufficient, to be re-repeated if there is no improvement in from eighteen to twenty-four hours: a third dose at a like interval may be necessary. For severe cases in children under two years and in mild cases an initial dose of 1,000 units is usually enough: it is to be repeated if necessary.

The Metropolitan Asylums Board have issued a statistical report[1] on the antitoxin treatment in six fever hospitals in 1895, as compared with cases treated in 1894, in which no antitoxin was used, and the mortality was exceptionally low. The general percentage mortality rate is decreased 7.1 in favour of 1895, and in cases in the first five years of life 13.2. The mortality when the treatment was begun on the first and second days of the disease is 4.6 and 14.8 per cent. respectively in 1895, as against 22.5 and 14.8 per cent. in 1894. The mortality in laryngeal cases is reduced from 70.4 per cent. in 1894 to 49.3 per cent. in 1895. The complications of the disease were apparently not influenced by antitoxin. The cases were 2182 in nuumber, and the authors lay particular stress on the importance of beginning the treatment as early as possible. They point out: (1) A great reduction in mortality in cases coming under treatment on the first or second day of the disease. (2) A general mortality below that of any other year. (3) A particularly remarkable lowering of mortality in laryngeal cases. (4) A uniform improvement in tracheotomy results. (5) A beneficial effect upon the clinical course of the disease.

Further points brought out in other reports are: the great importance of early treatment, some showing recovery of all cases injected on the first day of the disease; the early disappearance of the membrane, by the seventh day in over 80 per cent. of cases; the subsidence of severe laryngeal symptoms, and the smaller number of cases requiring tracheotomy: the Berlin report states that the mortality decreases as the age of the patient increases: other statistics show a striking decrease in the mortality in cases under five years of age.

Dr. Funck, of Brussels,[2] on the basis of the collection of a number of statistics, states: (1) Serum treatment diminishes the mortality of diphtheria. (2) Serum treatment often checks the

[1] Abstract in *Am. J. M. Sc.*, 1896, cxii. 225.

[2] *J. de Méd., Brux.*, 1896, No. 13; abstract in *Centralbl. f. innere Med.*, 1896, xvii. 1148.

further extension of the diphtheritic process, and avoids tracheotomy. (3) Sepsis, severe affections of the heart, kidneys, and nervous system are lessened. (4) The serum is absolutely harmless.

In a discussion at the New York Academy of Medicine,[1] Dr. W. H. Thomson gave the following statistics of the antitoxin treatment of diphtheria, drawn from a paper by Professor W. H. Welch in the *Johns Hopkins Hospital Bulletin* for July–August, 1895, and from a summary of the statistics of the treatment, brought up to May, 1896, by Dr. A. R. Guerard. Hospital reports are selected partly because they would not give the most favourable results, and therefore are less affected by the arguments brought forward by the opponents of the treatment. The reports are drawn from 85 hospitals in all the European countries, the United States, Australia, and Japan, giving a total of 9,893 cases with 1,820 deaths, or a mortality of 18.3 per cent. "In 53 of these hospital reports there are given statistics of 7,277 cases with a mortality of 20 per cent. under serum treatment in which definite comparisons are made with the mortality previous to the use of antitoxin of 44.3 per cent.; thus justifying the contention that so far as hospital practice goes antitoxin has caused a reduction of fully 50 per cent. in the general death rate." Further statistics from reports of 3,760 cases treated with antitoxin in private practice give 296 deaths, or 7.8 per cent.

A report from the Imperial Health Office in Berlin[2] for the second quarter, 1895, states that in 2,130 cases treated, excluding cases moribund on admission to hospital, the death-rate was 13.3 per cent. In the first quarter of the year the rate was 16.7 per cent. The injection was practised on the first two days of the illness in 752 patients, of whom only 48 died, a mortality of 6.4 per cent.; a much smaller death-rate than in the first quarter, due to the greater strength of the serum used. Injections performed on the third day gave a percentage mortality of 10; on the fourth day, 17.3; on the fifth day, 23.5; from the sixth to the tenth day, 28.3; from the eleventh to the nineteenth day, 17.6. 841 patients suffered from laryngeal diphtheria, of whom 588 were operated on, and 176 died; a mortality of 29.9 per cent. Secondary effects noted were, skin eruptions, pains in the joints, and suppuration at the site of injection.

In Glasgow,[3] in the years 1890–94, the percentage death-rate from diphtheria was respectively 39.8, 38.8, 37.2, 40.5, and in 1894, 35.5. In 1895, out of 179 cases 137 were treated with antitoxin, four being moribund on admission, and thirty-eight so mild as to give rise to no anxiety. Thus the severe cases only were treated by antitoxin. In seventy-six cases only one injection of from 10 to 20 c.c. was necessary; in the others additional ones were given. Of the total 179 cases twenty-five, or 14 per cent., died. That the fall in fatality is not due to a natural decline in

[1] *Med. Rec.*, 1896, xlix. 893. [2] *Lancet*, 1896, i. 331. [3] *Ibid.*, 1658.

severity of the disease in Glasgow is shown by the fact that the mortality in home-treated cases in 1895 was higher (24 per cent.) than in 1894 and 1893, 23 per cent. and 22 per cent. respectively. Of tracheotomy cases the fatality rate was in 1893, 76.2 per cent.; in 1894, 86.9 per cent.; and in 1895, only 34.5 per cent.

Dr. Virneisel reports[1] from the Coblentz Hospital 150 cases treated by antitoxin, of whom 9 died, or 12.7 per cent.: of 22 injected on the first day of the disease, all recovered; the death-rate for those injected up to the third day was 31.6 per cent.; after the third day, 68.4 per cent. Of 79 tracheotomy cases previous to the serum treatment 48 died, or a mortality of 61.4 per cent.; of 64 tracheotomy cases treated with serum 15 died, or a mortality of 23.4 per cent. Of the whole number treated, one-third were only brought to the hospital and injected on the fifth day of the disease; and of the 18 deaths, 6 patients were brought in a dying condition to the hospital. The author emphasises the great importance of early treatment.

Dr. Karl Fürth gives[2] the following experience at Freiburg:

| Date. | Cases treated: Without serum treatment. | Percentage of deaths. | Tracheotomy cases. | Percentage of deaths. |
|---|---|---|---|---|
| June, 1892—June, 1893 | 95 | 38.9 | 41.0 | 74.3 |
| June, 1893—June, 1894 | 145 | 38.6 | 52.4 | 67.1 |
| June, 1894—June, 1895 | 144 | 19.4 | 34.7 | 50 |
| | With serum treatment. | | | |
| June, 1895—June, 1896 | 150 | 15.3 | 23.3 | 42.8 |

Whilst in the earlier periods the death-rate for children under two years was 60—100 per cent. Of 19 cases under two years treated by serum only 3 died, although 8 required tracheotomy. In the later cases a larger dose of serum was employed—1690 units; in several cases 4000 units were administered within twenty-four hours. Of 46 cases treated on the first or second day of the disease, all recovered.

A later communication[3] from the Caroline Children's Hospital in Vienna states that of 633 cases treated before serum 292 died, or 46.1 per cent.; of 200 treated with serum 34 died, or 17 per cent.

| | | No. of Cases. | Deaths. | Percentage mortality. |
|---|---|---|---|---|
| Of these cases (1) Cases in which the throat or the larynx was affected and not operated on | before serum treatment | 331 | 83 | 25 |
| | with serum | 159 | 11 | 6 9 |
| ,, ,, ,, Operated on for Croup | before serum | 302 | 209 | 69.2 |
| | with serum | 41 | 23 | 56 |

1,000 units were injected on admission; if after 24—36 hours no tendency to heal or signs of spreading were observed, a second injection was given. In 69 cases localised to the throat there was only 1 death. In 10 out of 28 cases of laryngeal croup symptoms of stenosis declined shortly after admission;

[1] *München. med. Wchnschr.*, 1896, xliii. 456. [2] *Ibid.*, 669.
[3] *Wien. med. Wchnschr.*, 1896, ix. 795.

1 died of heart-failure, 2 of pneumonia, six and ten hours after admission respectively; in 15 cases tracheotomy was performed, of which 6 died. Three cases of diphtheria showed signs of septic infection. They terminated fatally, the injection of serum having no influence on them. The membrane disappeared on or before the fourth day in 23 cases, on or before the sixth day in 29, on the seventh day in 8, later than the seventh day in 8. There was albuminuria in 28 cases; symptoms of heart-failure observed in 5. An urticaria-like rash was observed in 9 cases; scarlatiniform or erythematous in 7.

The importance of early treatment is also brought out in a series of 352 cases reported by Dr. Aaser.[1] The mortality in cases treated on the first day of the disease was *nil;* in those treated on the second day it was .9 per cent.; on the third day, 11.6; after the seventh day, 50. The death-rate in cases in which tracheotomy or intubation was necessary was 29.2 per cent. The author thinks that erythema as a consequence of the injection is less frequent if the serum is filtered through a Chamberland filter.

As to **the immunising power** of the serum, the Coblentz Hospital Report[2] states that in four out of 150 cases treated a second attack occurred within twelve months of the first; and at the Caroline Hospital in Vienna relapses were noticed in several cases in a few days—2 to 18—after the first attack treated by antitoxin, and one patient had three distinct attacks in seven weeks. Kassowitz states[3] that neither a severe attack of diphtheria nor the injection of a large number of "immunising units" of serum brings in man insusceptibility to the diphtheria poison. After larger or smaller immunising doses of serum the attacks of diphtheria are as frequent as without them; the "immunised" person may suffer from an attack of diphtheria as soon as a few weeks or months afterwards, and the attack may be a severe one and end fatally in spite of serum-treatment. Rubens says[4] that a single injection of 200 units of Behring's serum should protect against diphtheria for several months. In the case, however, of a child affected with severe diphtheria, a brother aged 5 years, and a sister aged 7, were injected with 200 units as a protective measure. Four weeks later the brother passed through a typical attack of the disease, though not so severe as the first case.

With regard to any **ill effects** that may result from the antitoxin injections, it is obviously difficult to say what effects are due to the serum and what to the disease, or in fatal cases to know whether death is actually due to serum. Prof. Kolisko,

[1] *Tidsskr. f. d. norske Lægefor.*, 1896, Jan.; abstract in *Centralbl. f. innere Med.*, 1896, xvii. 923.

[2] *Loc. cit.*

[3] *Wien. med. Wchnschr.*, 1896, Nos. 21—23; abstract in *Centralbl. f. innere Med.*, 1896, xvii. 1145.

[4] *Deutsche med. Wchnschr.*, 1895, No. 46; abstract in *Centralbl. f. innere Med.*, 1896, xvii. 925.

of Vienna,[1] who had made 1000 autopsies on fatal cases of diphtheria before the serum treatment was introduced, considers, as a result of his *post-mortem* examinations on fatal cases treated by antitoxin, that the serum favourably influences the diphtheritic process; that as a consequence of it, where a sufficient dose has been given and an interval of not less than twenty-four hours has elapsed before death, the membrane is more easily separated, is looser, or pultaceous, whether in the pharynx, larynx, or bronchi, and that the anatomical changes in the organs are the same as those under the ordinary treatment. Perhaps more definite information can be gained from the use of the serum as a preventive measure, and the unfortunate results that have occurred in such cases show that Behring's original statement that the injection of his serum was as harmless as one of normal saline solution was too absolute. There is the much debated case of Prof. Langerhans's little boy, who died suddenly after a dose of antitoxin injected as a precautionary measure, the immediate cause of death not being satisfactorily accounted for. Other cases are given by Dr. Joseph Winters.[2] A healthy boy of 5 years was injected with a preventive dose of Behring's serum, and death occurred in five minutes (Halderman). A woman injected similarly with 600 units suffered from the most severe collapse for six hours. A boy aged 2 with spastic spinal paralysis, suffered from diarrhœa for four days after a preventive injection of 600 units, and died from collapse on the tenth day (Johannessen). A girl, in good health, aged 3, after an injection of 3 c.c., had a temperature of 104°, followed by albuminuria, hæmaturia, and a petechial rash, and died on the twentieth day (Alfoldi). In a girl aged 6, with slight angina, proved not to be diphtheritic, after a single injection the temperature rose to 105° on the sixth day, a scarlatiniform eruption appeared for the following four days, and death occurred in convulsions (Moizard and Bouchard). In some other cases severe symptoms followed preventive inoculations. Pistor[3] injected a girl aged 7, who suffered from tonsillitis in which diphtheria bacilli were absent, with 900 units of a serum, with the result that she suffered from a very protracted and variable illness, attended with fever with many remissions and exacerbations, and accompanied by exanthemata, the last of which appeared three months after the injection. These unfortunate results would appear to necessitate caution in the use of antitoxin as a preventive measure.

Drs. A. Seibert and F. Schwyzer,[4] however, as a result of experiments in the laboratory state: (1) That antitoxin serum does not seem capable of causing threatening symptoms or

[1] Quoted by Dr. Brannan, *Med. Rec.*, 1896, xlix. 900.

[2] Discussion at the New York Academy of Medicine. *Ibid.*, 877.

[3] *Deutsche Ärzte-Ztg.*, 1895, No. 24; abstract in *Centralbl. f. innere Med.*, 1896, xvii. 925.

[4] *N. York M. J.*, 1896, lxiii. 708; abstract in *Am. J. M. Sc.*, 1896, cxii. 216.

speedy death, even when brought quickly into the blood-stream in very large doses; (2) The carbolic acid used as a preservative must be in so weak solution as to be unable to cause the characteristic carbolic convulsions; (3) Even very small quantities of air, if injected, will cause severe disturbance and ultimate cessation of breathing, and to this cause the authors attribute the sudden deaths reported.

Dr. Winters[1] spoke unfavourably of the antitoxin treatment. He believes that in certain individuals there is a special susceptibility to antitoxin, grave symptoms or death resulting from a relatively small dose. The predominating symptoms are referable to the nervous centres, kidneys, heart, lungs, and the temperature. One feature of these symptoms, especially the pulmonary, is their late appearance. He brought forward forty-one fatal cases of diphtheria from the Willard Parker Hospital, in which antitoxin treatment was begun early in the disease, and had, in his opinion, an unfavourable influence. It is but right to add that in the same discussion Dr. Brannan, also of the Willard Parker Hospital, combated Dr. Winters's statements, stating that no special form of pneumonia had been observed, nor any *post-mortem* changes in fatal cases that could be attributed to antitoxin.

Dr. J. S. Billings, Jr.,[2] found in moderate and severe cases of diphtheria the blood corpuscles reduced in number, and only slowly regenerated. Leucocytosis occurs, except in very mild and in very severe cases, the increase in number of leucocytes being generally in proportion to the severity of the case. The percentage of hæmoglobin varies with the number of red corpuscles. In cases treated with antitoxin the decrease in the number of red corpuscles and in the amount of hæmoglobin is less than in cases not so treated. The leucocytosis is not affected. In healthy persons the injection of antitoxin causes in about half the cases a very moderate reduction in the number of red corpuscles and of hæmoglobin, and no change in the leucocytes. No peculiar characteristic changes in the morphology of the corpuscles were made out.

The diphtheria antitoxin has also been tried in **scarlet fever**, thus: J. Noir[3] injected 10 c.c. diphtheria serum into a child of 8 years on the third day of an attack of scarlet fever, with severe croupous inflammation of the fauces. The throat quickly improved: desquamation began on the third day after the injection. The chief point of the case lies in the absence of albumen in the urine, and as this occurred in a case of scarlet fever, the author infers that the serum may be used without any fear of irritating the kidneys. Dr. Szegö[4] treated a case of

[1] *Loc. cit.* [2] *Med. Rec.*, 1896, xlix. 577.

[3] *Progrès méd.*, 1895, 3e Sér. ii. 145; abstract in *Centralbl. f. innere Med.*, 1896, xvii. 880.

[4] *Deutsche med. Wchnschr.*, 1895, No. 51; abstract in *Rev. de Thérap. méd.-chir.*, 1896, lxiii. 10.

scarlet fever sore throat of the gravest kind with Behring's serum. Amelioration took place in twenty-four hours, followed by a rapid cure. Bacteriological examination showed the presence of staphylococci and streptococci, but not of Löffler's bacillus. The author invokes Metschnikoff's theory, increased phagocytosis, due to the stimulating effect of the antitoxin, to explain his case.

Injections of **anti-streptococcus serum** have been employed in scarlet fever, in erysipelas, puerperal fever, and acute septicæmia. This treatment must be considered as still on its trial, as it has not yet been employed in a sufficient number of cases to pronounce on its positive value. According to T. J. Bokenham,[1] "Antistreptococcus serum is serum obtained from the blood of an ass which has received during several months repeated and increasing injections of living virulent streptococci. It will be seen at once, therefore, that the principle involved differs in important respects from that involved in the preparation of antidiphtheria serum. In this last it is the toxins, formed by diphtheria bacilli in bouillon cultures, which are employed to set up immunity in animals furnishing the serum. Antistreptococcus serum . . . . may be expected to possess antimycotic rather than antitoxic properties."

Dr. Alexandre Marmorek, seeing the invariable presence in **scarlet fever** of complications due to infection by streptococci, has treated the disease with anti-streptococcic serum. From October 16th to December 31st, 1895, 103 children suffering from scarlet fever entered the Hôpital Trousseau. Seven of these cases were not treated by the serum, because they were in the desquamative stage. Of the remaining ninety-six cases a bacteriological examination showed in all the presence of the streptococcus, either alone or associated with other microbes. In seventeen other cases Löffler's bacillus was also found. Four of these last cases, which were admitted with symptoms of diphtheria, died in spite of treatment by both the streptococcic and antitoxic serums. All the children received on admission an injection of 10 c.c. of antistreptococcic serum, which was doubled if the general condition was serious. Treatment was confined to serum injections and antiseptic washes for the throat. The injections were repeated daily until the temperature fell. If swollen glands or albuminuria appeared the injections were resumed, and continued until the condition had returned to the normal. In ordinary cases 10 c.c. to 30 c.c. were injected; in more serious ones 40, 60, 70 c.c., and in one child of 4, with scarlatinal rheumatism, who recovered completely, 90 c.c. The most striking effect was on the swollen glands; in nineteen cases with this complication, resolution occurred in all without suppuration. In four children who

[1] *Brit. M. J.*, 1896, ii. 4.

[2] These quantities represent the total amount injected during the illness.

were admitted with suppurative otitis, the serum promptly stopped the purulent discharge. The advantages claimed for this treatment are—the prevention of grave complications, the rapid disappearance of false membranes, relief of delirium, improvement of the general condition, and a fall of temperature if fever be caused by complications due to streptococci. The fever caused by the scarlet fever virus pursues its ordinary course, as does the eruption, supporting the view that the disease is not caused by the streptococcus.[1] A later report says that Marmorek has treated 411 patients with a mortality of 3.4 per cent.

Baginsky[2] said he had used the serum of Marmorek in fifty-seven cases, from which nine must be deducted as the treatment was not fully carried out. In twenty-seven cases a fall of fever followed the injection, though, as this is sometimes observed in cases not so treated, it cannot be directly attributed to the serum. The sore throat and enlarged glands yielded in from three to five days, inunctions of iodine ointment being also used for the latter. In sixteen cases the serum treatment was entirely inefficacious. Some cases died from intensity of scarlet fever poison, in spite of the injection of maximum doses of the serum. In other less serious cases, suppuration of the cervical glands occurred. There remain five cases in which single injections were employed merely to combat complications, and with good results. The total mortality during the serum treatment was 14 per cent., instead of 22—24 per cent. in the previous period, too small a difference on which to base a favourable conclusion.

Dr. Dubois of Lille[3] also finds that injection of the serum causes an abrupt fall of the temperature if the fever be due to the streptococcus and not to scarlet fever poison. A dose of 10 c.c. is sufficient. The throat cleans rapidly, glandular swelling and otitis are relieved, and he considers that albuminuria may be prevented by use of the serum from the first.

M. Josias[4] has used two kinds of anti-streptococcic serum; one prepared from the sheep, the other from the horse. In the first series 49 children were injected with an average dose of about a drachm without accident, save some urticarial eruptions. In the second series 96 children received from two to eighteen drachms, resulting in various complications, among which were streptococcic abscesses, lymphangitis, and polymorphous eruptions. With sheep-serum the mortality was 2.08 per cent.; with horse-serum, 5.31 per cent.; with ordinary treatment as before serum it was 5.81 per cent. There were no serious results. He con-

[1] *Ann. de l'Inst. Pasteur*, 1896, x. 47.
[2] *Med. Week*, 1896, iv. 143.
[3] *Bull. méd. du Nord*, April 10, 1896; abstract in *Rev. de Thérap. méd.-chir.*, 1896, lxiii. 368.
[4] *Presse méd.*, 1896, No. 42; abstract in *Am. J. M. Sc.*, 1896, cxii. 470.

siders that the serum apparently acts upon streptococci, which do not cause suppuration.

On the other hand, Dr. Variot[1] found in ten cases of children affected with **membranous tonsillitis,** in whom he injected 10—20 c.c. of the serum, the results so grave that he has abandoned its use. The temperature was raised two or three degrees for some hours, the children became prostrate with dry tongue, and an abscess formed in the abdominal wall even when the injections were made with due antiseptic precautions.

M. Comby also states[2] that the mortality of this disease treated by Marmorek's serum was 8 per cent., whilst in the same pavilion the previous mortality was 5 per cent. Not only did the anti-streptococcic serum fail to lower the mortality, but it did not prevent the complications due to the streptococcus.

Prof. Petruschky[3] states that his repeated experiments of Marmorek's anti-streptococcic treatment do not confirm the statements given by the latter, and that there is yet no certain proof of the possibility of a serum-treatment against streptococcus infection. No protective influence could be obtained against "Marmorek's streptococcus" nor against two equally virulent organisms cultivated by Petruschky, either with Marmorek's serum or with that prepared by Niemann of Lyons after the method of Marmorek. He also, as the result of experiments on animals, advises against the practical application of the serum.

M. Deléarde,[4] of the Institut Pasteur de Lille, on the use of Marmorek's anti-streptococcic serum in **erysipelas**, says, first, that it is non-toxic, and has been used in new-born infants without any harm; it may be used in large doses. In erysipelas, as soon as the diagnosis is made, 10 c.c. of the serum are to be injected into the subcutaneous cellular tissue of the flank: benign cases are generally cured by one dose; if not, a second dose is injected after an interval of twenty-four hours. The temperature and the pulse are the guides to the need for repeating the dose. The effects of the serum show themselves from the fifth to the twelfth hour in a fall of temperature and pulse-rate, relief of headache, disappearance of albumen from the urine, and in improvement of the local signs. Occasionally, twelve hours after injection, a little pain, redness, and sometimes œdema, appear at the site of puncture, but as a rule these pass away without any trouble. Two or three hours after the injection there is a rise of temperature, which rapidly falls

[1] *J. de Clin. et de Thérap. inf.*, 1896, No. 23; abstract in *Rev. de Thérap. méd.-chir.*, 1896, lxiii. 368.

[2] *J. des Praticiens*, 1896, No. 20; abstract in *Am. J. M. Sc.*, 1896, cxii. 216.

[3] *Centralbl. f. Bacteriol. u. Parasitenk.*, 1896, Bd. 20, No. 4/5, *& Ztschr. f. Hyg. u. Infections-krankh.*, 1896, Bd. 22, No. 3; abstract in *Wien. klin. Wchnschr.*, 1896, ix. 877.

[4] *Nord. méd.*, 1896, iii. 17; abstract in *Rev. de Thérap. méd.-chir.*, 1896, lxiii. 332.

again. Desquamation begins early and is in large shreds. As said above, when the case is mild and injections are begun early, a single one is sufficient; if it is further advanced the fever yields more slowly, and cases of ambulant erysipelas usually need repeated injections.

At the Vienna Medical Society, Prof. Chrobak[1] described three cases in which anti-streptococcic serum was employed. The first case was one of erysipelas in a lying-in woman, who recovered in three days. In the second case symptoms of sepsis came on in a woman after operation for myoma; 30 c.c. of serum were injected within two days, after which her condition improved. In the third case, one of puerperal fever, 25 c.c. were injected in three days: the fever and rigors continued until after the last injection; but on the next day the fever rapidly decreased. Prof. Chrobak has not formed a definite opinion as to the value of the injections, but believes them to be harmless.

M. Bolognesi[2] criticises the results obtained by Chantemesse in the serum-treatment of erysipelas. He points out that many cases are remarkably mild, and that the mortality varies with the time of the year and the form of the affection present. He gives an account of 1000 cases at the Hôpital d'Aubervilliers, in which the total mortality was 3.5 per cent. In cases under different forms of treatment, but with no very bad ones, the mortality was .9 to 1.2 per cent. These last may be compared with those of M. Chantemesse, in which the mortality was 1.7 per cent. in those treated by *sérum efficace*, and was 1.03 per cent. in those treated by *sérum très efficace*. The general result of the serum-treatment of erysipelas gives a mortality of 2.59 per cent., and may be compared with the general mortality of the disease varying from 2—4 per cent. He considers that the serum-treatment, to be properly tested, must be tried on acute, severe cases, the mild ones being rejected.

Anti-streptococcic serum has been used in **puerperal fever.**[3] Dr. Ledrain[4] has recently reported a case in which the condition on the ninth day after delivery was as follows: Temperature 104°, pulse 148, small, compressible, tympanites and tenderness of abdomen, fœtid diarrhœa, dryness of tongue, and quiet delirium. 5 c.c. of anti-streptococcic serum, obtained from the Institut Pasteur, were injected. Next day the temperature had fallen to 101.3°, the pulse to 120. Six injections of 5 c.c. each time were made daily for five days, then an interval of a day was left and the last injection given. On the day following the third injection the temperature was 104°, the pulse 130, and there was some redness and swelling at the site of the first injection; the next day this had disappeared, and the patient made an uninterrupted and complete recovery.

[1] *Lancet*, 1896, i. 75.

[2] *Progrès méd.*, 1896, 3e Sér. iii. 106.

[3] See *Bristol M.-Chir. J.*, 1895, xiii. 139. [4] *Progrès méd.*, 1896, 3e Sér. iii. 227.

Dr. McKerron[1] reports three cases of puerperal fever thus treated. The worst case died, the others recovered. In the former four injections of 10 c.c. were given; in the two latter, three and two injections respectively: the injections were made into the arm, and in one of the successful cases tenderness at the site of injection and an erythematous rash over the limb were noted; no other symptoms due to the injections were observed. The author thinks that the good effects of the serum were shown in improvement of the pulse and subjective condition of the patients.

Messrs. Ballance and Abbott report[2] a case of **acute hemorrhagic septicæmia** treated by anti-streptococcus serum. The patient, a medical man, pricked his thumb whilst making a *post-mortem* examination on a case of suppurative peritonitis. Pain and swelling of the thumb began the same evening. The day after the temperature was 103° F.; there was a scarlet septic erythema over the whole body, the pulse was rapid and soft, and there was drowsiness, with shooting pains in the arm. Next day the condition was worse, the temperature being 104.7°, the pulse 150, with rapid respiration and increased drowsiness. 3.5 c.c. of anti-streptococcus serum (Burroughs, Wellcome & Co.) were injected every four hours; after eight injections, 7 c.c. were used; twenty-eight injections were made in all. The patient gradually recovered, the temperature reaching the normal on the twelfth and thirteenth days. The effect of the serum was shown in that (1) the mind became clear in spite of high fever; (2) frontal headache ceased; (3) the tongue began to clean; (4) the pulse became slower and stronger and the respiration slower; (5) the skin became moist; (6) the wounds healed without suppuration.

Two cases of blood-poisoning, in which the symptoms were severe, were treated by injections of anti-streptococcic serum by Messrs. A. H. and A. R. Cook[3] with good results; in the second case the effect of the serum is doubtful, as the injections were not made until three weeks after the local infection.

Another case of recovery from acute septicæmia after injections of anti-streptococcic serum is reported.[4] Mr. L. Strangways Hounsell relates[5] the case of a boy in whom a tooth was removed for alveolar abscess, and who was taken shortly afterwards with severe symptoms of septicæmia. His temperature was 104.5°, the pulse 156; at first delirious, he shortly afterwards became unconscious, with loss of control over micturition and defæcation. He was treated with injections of anti-streptococcic serum, with remarkable results; very soon after the injections the boy got better, and ultimately completely recovered.

Dr. W. H. Weaver[6] has used anti-streptococcus serum in the

[1] *Brit. M. J.*, 1896, ii. 1033. [2] *Ibid.*, 2. [3] *Ibid.*, 1315. [4] *Ibid.*, 647.

[5] *Clin. J.*, 1896, ix. 46. [6] *J. Am. M. Ass.*, 1896, xxvii. 542.

treatment of three advanced cases of **pulmonary phthisis**, with the result of reducing the pyrexia and the amount of expectoration.

J. MICHELL CLARKE.

In a measure confirmatory of the recent discovery of bacilli in **rheumatoid arthritis**, as recorded in the *Lancet* of April 25th, 1896, are the observations of MM. Chauffard and Ramond.[1] In a case of undoubted rheumatoid disease they found micro-organisms presenting the characteristic appearances of diplo-bacilli, short and thin, and which they state resemble the diplococci of Talamon. The organisms were found in the synovia and stained easily with all the ordinary reagents, but cultivation and inoculation experiments failed, as was possibly to be expected from the difficult nature of the organism and from the scarcity of material at their command.

Schüller,[2] who has also described an organism in this disease, mentions in his more recent writings the discovery of another micro-organism having many of the characteristics of the bacillus prodigiosus, and one therefore more nearly resembling that described in the *Lancet* than did the previous one.

G. A. BANNATYNE.

